# Retrospective Analysis of Predictive Biomarkers of Survival in Acute Exacerbation of Fibrosing Interstitial Lung Disease: A Single-Center Study in Spain

**DOI:** 10.3390/jcm14061974

**Published:** 2025-03-14

**Authors:** Antía Ferreiro-Posse, Galo Granados, Sara Salvador, Maria Florencia Pilia, David Espejo, Christian Romero, Iñigo Ojanguren, Xavier Muñoz, Ana Villar

**Affiliations:** 1 Department of Respiratory Medicine, University Hospital of Santiago de Compostela, 15706 Santiago de Compostela, Spain; antia.ferreiro.posse@sergas.es; 2 Department of Respiratory Medicine, University Hospital Vall d’Hebron, 08035 Barcelona, Spain; galodavid.granados@vallhebron.cat (G.G.); sara.salvador@vallhebron.cat (S.S.); maria.pilia@vhir.org (M.F.P.); david.espejo@vallhebron.cat (D.E.); christian.romero@vallhebron.cat (C.R.); inigo.ojanguren@vallhebron.cat (I.O.); xavier.munoz@vallhebron.cat (X.M.); 3 Vall d’Hebron Institut de Recerca (VHIR), Vall d’Hebron Barcelona Hospital Campus, 08035 Barcelona, Spain; 4 Spanish Biomedical Research Networking Centre (CIBERES), Carlos III Health Research Institute, 28029 Madrid, Spain

**Keywords:** fibrosing interstitial lung disease, acute exacerbation, corticosteroids, ischemic heart disease, survival

## Abstract

**Background**: Fibrosing interstitial lung diseases can evolve into acute exacerbations, which significantly impact morbidity and mortality. Currently, no routinely used clinical biomarkers can discern the potential progression in these patients. This study aims to analyze different biological markers used in routine clinical practice as possible predictive biomarkers for patients with acute fibrosing interstitial lung disease exacerbation. **Methods**: We conducted a retrospective, single-center study including patients diagnosed with acute exacerbation of fibrosing interstitial lung disease who required hospitalization between 2018 and 2019 at Vall d’Hebron Hospital, Spain. Patient demographics, clinical data, respiratory function, and comorbidities were collected at baseline. The primary outcome was survival at 30 days, 90 days, and 365 days, using Kaplan–Meier survival analysis and Cox regression. **Results**: Twenty-nine patients were included (mean age 70.4 years). At the 3-month follow-up, patients with ischemic heart disease showed higher survival rates (*p* = 0.02). Identifying an infection as the etiology of the exacerbation was associated with worse one-year survival rates compared to idiopathic cases (*p* = 0.03). Elevated levels of leukocytes (*p* < 0.01), neutrophils (*p* < 0.01), and fibrinogen (*p* = 0.03) were predictors of mortality. Additionally, patients who received a cumulative dose of corticosteroids between 501 and 1000 mg during the exacerbation showed higher one-year survival (*p* < 0.01). **Conclusions**: Routine clinical markers can help predict outcomes in AE-f-ILD. Further multicenter studies should validate these findings and assess the role of therapies in its management.

## 1. Introduction

Fibrosing interstitial lung diseases (f-ILD) constitute a heterogeneous group of disorders characterized by involvement of the alveolar–interstitial space, leading to progressive fibrosis, disorganization of the lung parenchyma, and chronic respiratory failure [1,2,3,4,5,6,7]. Decline of FVC, worsening of symptoms, and evidence of radiologic progression define the fibrotic phenotype in ILD and lead to the initiation of antifibrotic treatment [7].

The prevalence of the progressive fibrosing phenotype in non-IPF fibrotic ILDs varies depending on the studies, ranging between 25% and 35% [8,9]. In some cases, the disease course can be insidious, manifesting as an acute exacerbation of fibrosing interstitial lung disease (AE-f-ILD), which significantly impacts morbidity and mortality. Although AE-f-ILD was initially described for idiopathic pulmonary fibrosis (AE-IPF), an international working group recently proposed classifying AE-f-ILD into two major categories: those with an identifiable trigger (infection, post-surgical procedure, toxicity, or aspiration) and idiopathic cases where no trigger is identified, thus removing the requirement for idiopathic etiology from the definition. Cases with an extra-parenchymal cause, such as heart failure, pneumothorax, pleural effusion, or pulmonary thromboembolism, are excluded from the AE-f-ILD definition [10].

Other progressive fibrosing interstitial lung diseases (f-ILD) in addition to IPF can exhibit a similar clinical course [11,12,13]. However, there is no established definition for this subgroup of patients with AE-f-ILD. In these cases, as in IPF, it is sometimes possible to identify the causal trigger of respiratory failure, while in other instances, the cause remains idiopathic [10]. Regardless of etiology, AE-f-ILD, including IPF, is characterized by diffuse alveolar damage as the predominant pattern. However, other histological patterns can also be observed, such as organizing pneumonia, alveolar hemorrhage, or nonspecific inflammatory changes [10,14].

Regarding the treatment for AE-f-ILD, the most accepted recommendation (weak recommendation) is the administration of high-dose corticosteroids, with inconsistent efficacy data according to available studies [15]. Other therapeutic options described for refractory cases include cyclosporine, cyclophosphamide, polymyxin B hemoperfusion, rituximab, immunoglobulin infusion, or plasmapheresis, all with low scientific evidence [16,17,18]. For a percentage of patients, lung transplantation is the only therapeutic alternative. Still, it is not always a feasible strategy [19], with acute exacerbation being an independent poor prognostic factor for mortality in transplanted patients [20].

In routine clinical practice, no prognostic markers are available to predict the evolution of patients with AE-f-ILD or potentially base preventive interventions on selected cases. However, there are promising data on various biomarkers in IPF used at the research level, such as epithelial cell markers (KL-6, SP-A, SP-D), immunological dysregulation markers (CCL18, YKL-40), extracellular matrix remodeling and fibrogenesis markers (MMP-7), and the determination of circulating fibrocytes, which are currently being evaluated for other f-ILDs, although their use in routine practice is anecdotal [21,22,23,24].

Having prognostic information can be helpful in therapeutic decision making and follow-up of these patients [16]. Therefore, this study aims to evaluate different biological markers used in routine clinical practice and their prognostic value in cases of AE-f-ILD.

## 2. Materials and Methods

### 2.1. Design and Participants

This is a retrospective, single-center observational study analyzing data from patents with fibrosing interstitial lung disease (f-ILD) diagnosed with acute exacerbation (AE-f-ILD) with clinically significant respiratory deterioration, requiring hospitalization in 2018 and 2019 at the Pulmonology Service of Vall d’Hebron Hospital. Patients were initially assessed in the hospital’s emergency department by an on-call pulmonologist, who diagnosed AE-f-ILD and determined the need for admission to either the respiratory department or the intensive care unit.

Inclusion criteria [1,10,14,25]: (1) Known or concurrent diagnosis of f-ILD; (2) acute worsening or development of dyspnea of less than one month’s duration; (3) new bilateral ground-glass opacities or consolidation superimposed on a pattern compatible with fibrotic lung disease on computed tomography; and (4) deterioration not explained by heart failure, pulmonary embolism, or fluid overload.

### 2.2. Variables Studied

Demographic and Clinical Variables: gender, age, tobacco consumption, cumulative exposure, and associated comorbidities quantified using the Charlson index. Forced vital capacity (FVC) is expressed as percentages of predicted average values recorded in the last forced spirometry performed before the exacerbation.

Chest tomography, the presence of the usual interstitial pneumonia (UIP) pattern or other specific ILD patterns in previous studies in previous studies, and the percentage of ground-glass opacities during the exacerbation were evaluated.

Analytical data collected included serum levels of leukocytes, platelets, D-dimer, lactate dehydrogenase (LDH), C-reactive protein (CRP), and interleukin-6 (IL-6).

We are identifying infectious triggers through microbiological isolation performed in sputum cultures, polymerase chain reaction (PCR) for respiratory viruses, blood cultures, serologies for atypical respiratory pathogens, and urine antigen tests for pneumococcus and Legionella.

Additionally, the degree of respiratory failure on admission was determined using the PAFI scale, defined as the ratio of arterial oxygen pressure to inspired oxygen fraction (PaO_2_/FIO_2_). This allows the assessment of oxygenation and the severity of respiratory distress. It is subdivided into three categories: mild (PAFI > 200 but ≤300) (level 1), moderate (PAFI > 100 and ≤200) (level 2), and severe (PAFI ≤ 100) (level 3) [26]. The type of maximum invasive or non-invasive respiratory support received will also be recorded.

**Figure 1:**
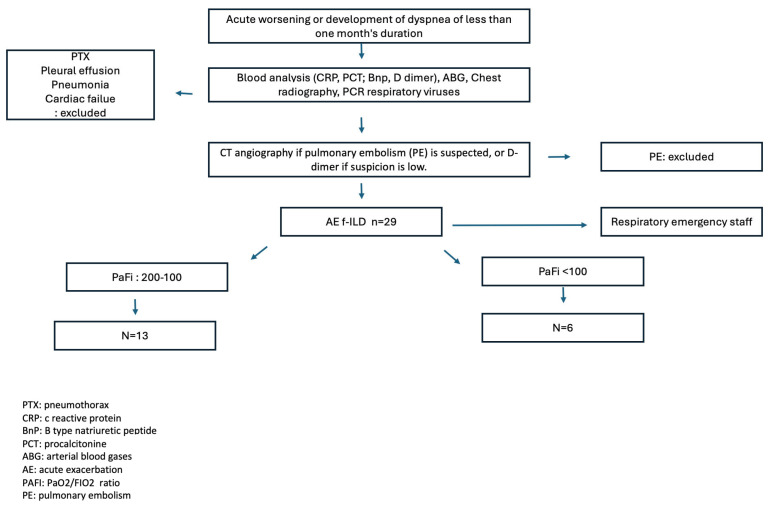
Cohort diagram.

To evaluate corticosteroid treatment received for exacerbation, the equivalent dose of cortisone was used, divided into four groups: ≤500 mg; 501 to 1000 mg; 1001 to 1500 mg; and ≥1501 mg, coinciding with the quartiles of our sample distribution. Additionally, the cumulative dose of corticosteroids in mg received during hospitalization was assessed. Notably, for 5 of the 29 patients included in this study, the cumulative cortisone dose could not be determined as they were admitted to the intensive care unit, where the prescription application was inaccessible (Figure 1).

### 2.3. Statistical Analysis

Results are expressed as absolute numbers and corresponding percentages for qualitative variables, mean and standard deviation (SD) for quantitative variables with normal distribution, and median and 25th to 75th percentiles for quantitative variables with non-normal distribution. Comparisons between groups were made using the chi-square test with Fisher’s exact test for qualitative variables and Kruskal–Wallis for quantitative variables, given the sample size. Survival analyses were conducted with 30-, 90-, and 365-day cutoffs and Kaplan–Meier curves for qualitative variables; survival differences for other variables were assessed using univariate Cox regression. The Spearman correlation test was used to study the relationship between baseline PAFi and the cumulative corticosteroid dose. The analysis used Stata IC 14 (StataCorp. 2015. Stata Statistical Software: Release 14. College Station, TX, USA: StataCorp LP). A *p*-value < 0.05 was considered statistically significant. 

This study was conducted according to the guidelines of the Declaration of Helsinki, and approved by the Research Ethics Committee of Vall d’Hebron University Hospital with code PR(AG) 99/2024 on date 27 September 2024.

## 3. Results

A total of 29 patients were included (7 IPF, 22 non-IPF), of whom 21 were males, with a mean age of 70.4 ± 8.4 years and a mean FVC of 65.4 ± 17.5% (62.7 ± 18.6% IPF, 66.1 ± 17.6% non-IPF) (Table 1).

Out of the total patients, 25 had a prior diagnosis of f-ILD. Regarding the radiological pattern, 25% of the cases were consistent with a UIP pattern. At the time of acute exacerbation, 17 patients (59%) were being treated with immunosuppressants, and 6 of them were receiving antifibrotic treatment. The subgroup analysis showed statistically significant differences in ESR levels (16.8 ± 11.4 mm/h IPF, 44.9 ± 32.2 mm/h non-IPF) (*p* = 0.04) and the percentage of ground-glass opacities on chest CT (33.3% IPF, 86.4% non-IPF) (*p* = 0.02) (Table 1).

Characteristics related to acute exacerbation are shown in Table 2.

The mean PAFi on admission was 132.33 ± 48.5 mmHg (124.0 ± 75.0 mmHg IPF, 133.3 ± 47.8 mmHg non-IPF), and in only 9 patients was it possible to identify the trigger for respiratory deterioration. Univariate Cox analysis revealed that leukocyte levels (HR 1.17; *p* < 0.01), neutrophil levels (HR 1.28; *p* < 0.01), and fibrinogen levels (HR 1.66; *p* = 0.03) were predictors of mortality.

Fifty percent of the patients received between 0.5 and 1 g of corticosteroids, and 93% were treated with HFNC (Table 3).

The mean cumulative corticosteroid dose was 854.8 ± 721.4 mg, with higher values for the non-IPF subtype (937.6 ± 785.5 mg) compared to IPF (540.0 ± 242.8 mg), without reaching a statistically significant difference (*p* = 0.27). In five patients, the cumulative corticosteroid dosage could not be determined due to lack of access to the specific hospital unit’s prescription software.

In most patients, the respiratory support used was high-flow nasal cannulas (HFNC) (26 patients). However, in three patients, non-invasive mechanical ventilation (NIMV) was chosen. When these two therapeutic measures failed, orotracheal intubation was the alternative therapy used in five patients.

The 3-month survival rate showed differences related to gender, being 63.69% (95%CI: 38.47–80.81) in males and 15% (95%CI: 0.75–47.94) in females (*p* < 0.01), as well as the presence of ischemic heart disease (IHD), being 77.78% (95%CI: 36.48–93.93) in the IHD group and 36.91% (95%CI: 15.47–58.72) in the group without IHD (*p* = 0.02) (Table 4).

**Figure 2:**
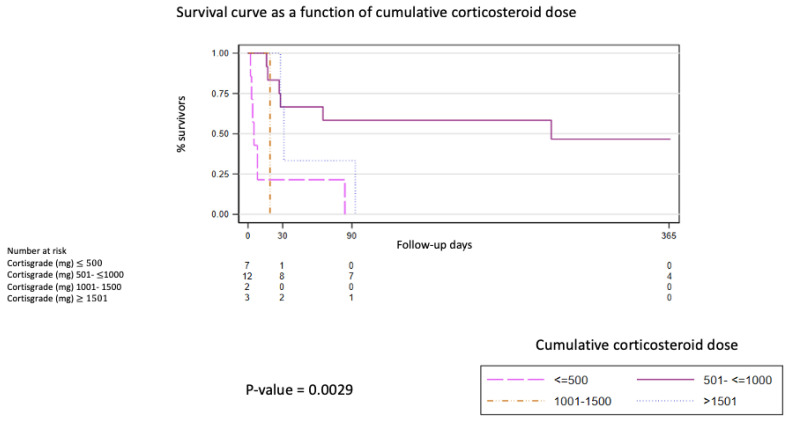
Survival curve as a function of cumulative corticosteroid dose.

Identification of the trigger was associated with poorer 1-year survival rates of 0% (95%CI: 0.61–38.77), compared to idiopathic cases of 32.86% (95%CI: 11.28–56.68; *p* = 0.03). It was also observed that patients who received a cumulative corticosteroid dose between 501 and 1000 mg showed higher 1 year-survival of 46.67%, (95%CI: 16.78–72.22; *p* = 0.0029), (Figure 2). A correlation analysis was performed between baseline PAFi and the cumulative corticosteroid dose, which did not yield a statistically significant result (*p* = 0.719).

## 4. Discussion

Our study retrospectively analyzed 29 patients with AE-f-ILD, evaluating different biological markers used in routine clinical practice and their predictive value during an acute exacerbation.

One of the most significant findings in our study was the higher survival rate in patients with treated ischemic heart disease. It is known that atherosclerosis is an inflammatory disease, and patients with acute coronary events exhibit systemic elevation of cytokines and procoagulant factors [27]. Similarly, it has been demonstrated that in cases of community-acquired pneumonia, an acute systemic inflammatory response can persist even after the resolution of the infection, which is associated with vascular events [28]. In both scenarios, platelet activation plays a significant role in the innate immune response, which may predispose to thrombotic and ischemic events [29,30]. Thus, cardiovascular event prevention strategies, particularly antiplatelet therapy, are recommended in both contexts [31]. Parallelly, in animal models of IPF, an imbalance between thrombotic and fibrinolytic phenomena in the alveolar space has been described, with a tendency towards a systemic prothrombotic state [32,33,34]. In this context, anticoagulant treatment has been proposed for individuals with IPF in the past; however, results from various studies have been controversial [35,36,37], and this therapy is not currently recommended for this patient profile.

Moreover, beyond the mentioned coagulation alteration, different studies have suggested that antiplatelet drugs such as acetylsalicylic acid or ticagrelor could have an antifibrotic effect on various organs (liver, heart, endometrium) [38,39,40]. During fibrogenesis, it has been observed that the primary pathway regulating the autophagy process, known as the phosphatidylinositol 3 kinase/protein kinase B/mammalian target of rapamycin (PI3K/AKT/mTOR) pathway, is altered [41]. Additionally, the significant pro-fibrogenic role of the cytokine TGF-β1 in idiopathic pulmonary fibrosis is well known, with platelet activation being an essential source of this cytokine. In this regard, treatment with acetylsalicylic acid has been shown to enhance the number of autophagosomes, thereby promoting autophagy through inhibition of the PI3K/AKT/mTOR pathway, a finding demonstrated in vitro. This could block fibroblast differentiation, reducing extracellular matrix deposition [42]. Based on our study results, we hypothesize that the antiplatelet treatment received by individuals with a history of treated ischemic heart disease might justify the need for prospective studies to specifically evaluate the role of antiplatelet therapy in this context.

Regarding identifying a trigger for respiratory failure to guide a specific treatment regimen, one would expect that its identification would improve the prognosis of patients with AE-f-ILD. However, our study found that detecting a trigger did not directly impact survival. This suggests that the role of the trigger in exacerbation is limited to activation but does not influence the subsequent perpetuation of the inflammatory and fibrogenic process. On the other hand, idiopathic cases reflect the absence of trigger identification with standard study methods. Although there are no standardized study protocols, other potential AE causes, such as pollution, are only sometimes assessed, potentially leading to a higher number of unidentified triggers that could alter the results. Further studies with a standardized early protocol to search for the cause of AE-f-ILD and compare survival in a larger patient sample based on trigger identification are needed.

Conversely, inflammatory parameters such as leukocytosis, neutrophilia, and blood fibrinogen levels were predictors of mortality in our patient series. This suggests that the degree of systemic inflammation, as measured by these serum markers, should guide clinical decision making regarding the severity and prognosis of acute exacerbation.

Additionally, we observed that a cumulative cortisone dose between 501 and 1000 mg was associated with higher survival rates. The 2011 guidelines of the American Thoracic Society/European Respiratory Society/Japanese Respiratory Society/Latin American Thoracic Association for the diagnosis and management of IPF recommended, based on expert consensus, that individuals with AE-IPF should be treated with corticosteroids [43]. However, there are no clinical trials to base both efficacy and dosage in individuals with AE-f-ILD. The pathophysiological aspects involved in AE-f-ILD need to be better understood. It is known that there is an initial activation of the inflammatory cascade with the appearance of hyaline membranes and interstitial edema, followed by a fibrosis pattern characterized by extracellular matrix deposition and lung remodeling [44]. Corticosteroid treatment was proposed for its anti-inflammatory and immunosuppressive effects. Both physiological and anti-inflammatory aspects are modified through gene expression mechanisms, leading to the well-known adverse effects associated with this therapy [45]. In individuals with f-ILD experiencing an acute exacerbation, whether triggered by a respiratory infection or idiopathic, initial activation of the innate immune response occurs, followed by an adaptive response, culminating in lung remodeling [46]. Corticosteroids, pending new studies that may change therapeutic management, remain the drugs of choice in managing AE-f-ILD cases. This study is the first to demonstrate a cumulative cortisone dose that improves survival in patients with AE-f-ILD.

The limitations of our study include the small sample size, limiting a proper evaluation of potential prognostic markers for AE-f-ILD, and the retrospective nature of this study without a standardized hospital care protocol for AE-f-ILD cases, which would allow a homogeneous assessment of the different variables. Our results are exploratory and require validation in studies with larger sample sizes and multivariate adjustments.

This study has several inherent limitations that should be considered when interpreting the findings. First, it is a single-center, retrospective study conducted without a standardized protocol for the hospital management of AE-f-ILD cases. This lack of standardization hinders the homogeneous assessment of the various analyzed variables. Second, the small sample size precluded the possibility of performing a multivariable analysis, thereby limiting the statistical power and the generalizability of the results.

## 5. Conclusions

In conclusion, this study demonstrates that measuring serum markers in routine clinical practice can help establish a prognosis for individuals with AE-f-ILD. Additionally, we propose that antiplatelet drugs may contribute to improved survival in individuals with a history of treated ischemic heart disease and AE-f-ILD. Furthermore, this is one of the first studies with a larger number of patients included that correlates a cumulative corticosteroid dose with improved survival in patients with AE-f-ILD. Although these findings require validation through future studies with larger sample sizes, the data presented provide valuable insights to optimize the clinical management of these patients and open new avenues for therapeutic research in this field.

## Figures and Tables

**Table 1:** General Characteristics of the Population.

Demographic Data	Total (n: 29)	Non-IPF (n: 22)	IPF (n: 7)	*p*-Value
Sex, male, n, %	21 (72.4%)	14 (63.6%)	7 (100%)	0.0608
Age, mean (SD)	70.5 (8.6)	69.1 (8.1)	74.9 (9.2)	0.1137
**Smoking status, n, %**				
Non-smoker	7 (24.1%)	7 (31.8%)	0 (0%)	0.1768
Active smoker	1 (3.4%)	1 (4.5%)	0 (0%)	
Ex-smoker	21 (72.4%)	14 (63.6%)	7 (100%)	
Charlson Index, mean (SD)	2.8 (1.7)	2.9 (1.9)	2.3 (1.1)	0.6365
**Prior f-ILD (n, %)**				
No	4 (13.8%)	4 (18.2%)	0 (0%)	0.5461
Yes	25 (86.2%)	18 (81.8%)	7 (100%)	
**Prior f-ILD diagnosis (n, %)**				
f-NSIP	5 (17.2%)			
f-HP	4 (13.8%)			
SRIF	3 (10.3%)			
IPF	7 (24.1%)			
CPFE	2 (6.9%)			
f-Drug-induced interstitial pneumonitis	2 (6.9%)			
f-Sjögren’s syndrome lung disease	1 (3.4%)			
f-COP	1 (3.4%)			
f-ILD sequelae of distress syndrome	1 (3.4%)			
f-Unclassifiable interstitial lung disease	3 (10.3%)			
**Spirometry, FVC (%), mean (SD)**	65.4 (17.5)	66.1 (17.6)	62.7 (18.6)	0.6440
**Prior chest CT (percent glass)**				0.0675
No	8 (27.6%)	3 (13.6%)	5 (71.4%)	
<25%	9 (31%)	8 (36.4%)	1 (14.3%)	
25–50%	5 (17.2%)	4 (18.2%)	1 (14.3%)	
50–75%	2 (6.9%)	2 (9.1%)	0 (0%)	
>75%	5 (17.2%)	5 (22.7%)	0 (0%)	
**UIP pattern present (n, %)**				<0.0001
No	21 (75%)	21 (95.5%)	0 (0%)	
Yes	7 (25%)	1 (4.5%)	6 (100%)	
**Laboratory data (mean, SD)**				
ESR	37.9 (30.8)	44.9 (32.2)	16.8 (11.4)	0.0444
LDL	537.7 (173.2)	549.4 (186.6)	507.2 (146.7)	0.9215
Treatments received (n, %)				
Prior immunosuppressants, n, %	17 (60.7%)	12 (54.5%)	5 (83.3%)	0.3547
**Prior immunosuppressants (types)**				
Mycophenolate		2 (16.7%)	1 (20%)	0.61026
Prednisone		5 (41.7%)	4 (80%)	
Methylprednisolone		2 (16.7%)	0 (0%)	
Mycophenolate + prednisone		3 (25%)	0 (0%)	
Prior antifibrotics		2 (9.1%)	4 (66.7%)	
**Prior LTOT (n, %)**				0.986
No		7 (31.8%)	2 (33.3%)	
Resting LTOT		14 (63.6%)	4 (66.7%)	
Exercise-induced LTOT		1 (4.5%)	0 (0%)	

Data are presented as the mean (m), standard deviation (SD), and percentages (%). Abbreviations: IPF, idiopathic pulmonary fibrosis; f-ILD, fibrosing interstitial lung disease; f-NSIP, fibrosing-Nonspecific Interstitial Pneumonia; f-HP, fibrosing hypersensitivity pneumonitis; SRIF, smoking-related interstitial fibrosis; IPF, idiopathic pulmonary fibrosis; CPFE, combined pulmonary fibrosis and emphysema syndrome; f-COP, fibrosing-cryptogenic organizing pneumonia; FVC, forced vital capacity; chest CT, chest computed tomography; UIP, usual interstitial pneumonia; ESR, erythrocyte sedimentation rate; LDL, lactate dehydrogenase; LTOT, long-term oxygen therapy.

**Table 2:** Characteristics Related to AE-f-ILD.

Data	Total	Subgroup		*p*-Value
		Non-IPF	IPF	
**Trigger (n, %)**	9 (33.3%)	7 (31.8%)	2 (40%)	1.0001
**PAFI (mean, SD)**	132.3 (48.5)	133.3 (47.8)	124.0 (75)	0.94695
**Severity level of PAFI (n, %)**				
2	13 (68.4%)	12 (70.6%)	1 (50%)	1.0001
3	6 (31.6%)	5 (29.4%)	1 (50%)	
**Laboratory (mean, SD)**				
ESR	37.9 (30.8)	44.9 (32.2)	16.8 (11.4)	0.0444
LDL	537.7 (173.2)	549.4 (186.6)	507.2 (146.7)	0.9215
Leukocytes × 10^9^	13.9 (4.0)	13.6 (3.8)	15.2 (5.1)	0.6144
Neutrophils × 10^9^	11.6 (3.6)	11.4 (3.6)	12.4 (3.6)	0.4495
Lymphocytes × 10^9^	1.3 (0.9)	1.2 (0.5)	1.7 (1.7)	0.9777
Platelets × 10^9^	277.0 (85.5)	284.5 (78.6)	250.7 (110.9)	0.4310
Fibrinogen	5.2 (1.1)	5.2 (1.2)	5.0 (0.9)	0.5946
D-dimer	866.8 (756.7)	662.4 (544.2)	1412.0 (1101.6)	0.1531

Data are presented as the mean (m), standard deviation (SD), and percentages (%). Abbreviations: AE-f-ILD, acute exacerbation of fibrosing interstitial lung disease; PAFI: PaO_2_/FiO_2_; ESR, erythrocyte sedimentation rate; LDL, lactate dehydrogenase.

**Table 3:** Treatments for AE-f-ILD.

Data	Subgroup		Total	*p*-Value
	Non-IPF	IPF		
**Cumulative Cortisone Dose (mg), (mean, SD)**	937.6 (785.5)	540.0 (242.8)	854.8 (721.4)	0.2707
**Cortisone Dose (mg), (n, %)**				
≤500	5 (26.3%)	2 (40%)	7 (29.2%)	0.967
501–≤1000	9 (47.4%)	3 (60%)	12 (50%)	
1001–1500	2 (10.5%)	0 (0%)	2 (8.3%)	
>1501	3 (15.8%)	0 (0%)	3 (12.5%)	
**Respiratory Support (n, %)**				
HFNC	20 (95.2%)	6 (85.7%)	26 (92.9%)	0.4445
NIV	2 (9.5%)	1 (16.7%)	3 (11.1%)	0.5454
IMV-IOT	4 (19%)	1 (16.7%)	5 (18.5%)	0.987
**Total**	22 (75.9%)	7 (24.1%)	29 (100%)	

Data are presented as the mean (m), standard deviation (SD), and percentages (%). Abbreviations: AE-f-ILD, acute exacerbation of fibrosing interstitial lung disease; IPF, idiopathic pulmonary fibrosis; HFNC, high-flow nasal cannula; NIV, non-invasive mechanical ventilation; IMV, invasive mechanical ventilation.

**Table 4:** One-Year Overall Survival of AE-f-ILD.

Clinical Data	N Subjects	N Deaths	*p*-Value
**Sex (n)**			
Female	8	7	0.0072
Male	21	12	
**Smoking Status (n)**			
Non-smoker	7	6	0.06
Active smoker	1	0	
Ex-smoker	21	13	
**History of Ischemic Heart Disease (n)**			
No	19	15	0.029
Yes	10	4	
**Prior Immunosuppression (n)**			
No	11	8	0.2402
Yes	17	11	
**Prior Antifibrotics (n)**			
No	22	14	0.3287
Yes	6	5	
**Use of LTOT (n)**			
No	9	6	0.0558
LTOT at rest	18	12	
LTOT only with effort	1	1	
**Trigger (n)**			
No	18	10	0.0291
Yes	9	9	
**Cumulative Cortisone Dose (n)**			
≤500	7	6	0.0029
501–≤1000	12	6	
1001–1500	2	1	
>1501	3	3	

Data are presented as the number of cases (n) and percentages (%), and confidence intervals to 95% (95% CI). Abbreviations: AE-f-ILD, acute exacerbation of fibrosing interstitial lung disease; LTOT, long-term oxygen therapy.

## Data Availability

The datasets analyzed in this study are available from the corresponding author on reasonable request.

## Abbreviations

The following abbreviations are used in this manuscript:

AE-f-ILD	Acute exacerbation fibrosing interstitial lung disease
AE-IPF	Acute exacerbation idiopathic pulmonary fibrosis
CAD	Coronary artery disease
CRP	C-reactive protein
f-ILD	fibrosing interstitial lung disease
FVC	Forced vital capacity
IPF	Idiopathic pulmonary fibrosis
LDH	Lactate dehydrogenase
UIP	Usual interstitial pneumonia
